# Disclosure of HIV test results by women to their partners following antenatal HIV testing: a population-based cross-sectional survey among slum dwellers in Kampala Uganda

**DOI:** 10.1186/s12889-015-1420-3

**Published:** 2015-01-31

**Authors:** Anthony Batte, Anne Ruhweza Katahoire, Anne Chimoyi, Susan Ajambo, Brenda Tibingana, Cecily Banura

**Affiliations:** Child Health and Development Centre, Makerere University College of Health Sciences, Kampala, Uganda; Ndejje University, Kampala, Uganda; University of Manchester, England, UK

## Abstract

**Background:**

Disclosure of HIV status by women to their partners is the backbone for prevention of HIV transmission among couples as well as promotion of the prevention of mother to child transmission of HIV interventions. The aim of this study was to determine the prevalence and factors associated with disclosure of HIV test results by women to their sexual partners following antenatal HIV testing in Kamwokya slum community, Kampala, Uganda.

**Methods:**

This was a population based cross-sectional study carried out from October to November 2011. A total of 408 randomly selected women aged 18–45 years, who had delivered a child within 2 years prior to the study, and had tested for HIV during antenatal care were recruited from Kamwokya community. A standardised interviewer- administered questionnaire was used to collect data. Data was entered into Epidata 2.1b and analysed using SPSS software version 16.0 and StatsDirect version 2.8.0.

**Results:**

Overall 83.8% (95% CI: 79.9- 87.1) of the women reported that they had disclosed their HIV status to their sexual partners. Disclosure was significantly higher among women whose partners had also tested for HIV (OR=24.86, 95% CI: 5.30 - 116.56). Other factors that were associated with disclosure were secondary education or above (OR=2.66, 95% CI: 1.34 - 5.30), having attended 3 or more antenatal care visits (OR=3.62, 95% CI: 1.70 - 7.72), being married/cohabiting (OR=8.76, 95% CI: 4.06 - 18.81) and whether or not they would opt not to disclose a family member’s HIV status (OR=1.61, 95% CI: 1.003 - 2.58). Overall, stigma was not significantly associated with disclosure.

**Conclusions:**

Disclosure of HIV test results to sexual partners in this group of women was relatively high. The results suggest that having a sexual partner who had also tested probably made it easier to disclose the woman’s HIV status. Other predictors of disclosure were secondary education and above and having attended more antenatal care visits. These findings suggest the need for promotion of sexual partner HIV testing, improvement of literacy levels of women, and encouragement of women to attend antenatal care, as key factors in promoting disclosure of HIV results.

## Background

Disclosure of HIV sero-status to sexual partners by pregnant women is key to prevention of transmission of HIV to partners and unborn children [1]. People are encouraged to disclose their results to their sexual partners because of the associated positive outcomes. Following disclosure of HIV results, the partner is more likely to: also test for HIV, disclose his/her HIV results, accept to implement HIV preventive behaviour for example use condoms, and more easily access care if HIV positive [2]. HIV positive women who disclose their results following testing during antenatal care (ANC) have increased emotional and financial support, freedom to use their HIV drugs before the person they disclosed to [3] and freedom to implement the feeding option they choose for their new-born [4].

Following disclosure of positive HIV results, while men are reportedly accorded acceptance, care and support by their partners; women are more likely to face discrimination by neighbours, friends and relatives [5]. Other negative outcomes that have been reported following disclosure of HIV results include: reduced social support [6], blame for testing without partner’s consent [7] and separation or divorce [8]. Regardless of whether women are HIV negative/positive, one study found that disclosure of HIV results is associated with intimate partner violence [9]. Consequently, the observation of these negative outcomes, the discrimination following disclosure [10], and the uncertainty of the partner’s reaction, have been cited as some of the reasons that prevent women from disclosing their results to their partners [11]. High stigma levels in a population have also been associated with non-disclosure of HIV results [4,12,13].

Over 60% of the urban population in Uganda lives in slums [14] and people living in African slums reportedly have HIV prevalence rates that are twice the rates of non-slum dwellers in a given city [15]. Women living in urban slums are particularly at risk of HIV as they reportedly have an early sexual debut, lower usage of condoms and more multiple sexual partners [16,17] compared to non-slum dwellers. The failure to disclose their HIV results may be due to fear of adverse outcomes of disclosure; which outcomes may affect their livelihood [18]. For instance, one group of women slum dwellers reported that if the current relationship did not work, it would not be possible to have another relationship with another man if the woman had disclosed her results to her partner [18]. Abandonment following disclosure may also result in loss of material and financial support especially if the woman is unemployed and relies on the sexual partner [19].

There has been a lot of work done to determine predictors of HIV disclosure between sexual partners. The predictors include: older age (>25 years vs < 25 years), being married or living with partner, higher education level and higher socioeconomic class [1,4,20,21]. Pregnant women are more likely to disclose their HIV status if: they are HIV negative, have few sexual partners (less than 6), know someone with HIV/AIDS, or know their sero-status before they become pregnant [21-23].

However, these studies were mostly conducted at health centres [1,21,24] or in rural communities [22]. Information about prevalence and predictors of disclosure among women living in slums is limited. Therefore, it was important to study disclosure factors among this high risk group (women living in the slums) regardless of their HIV status because majority of new heterosexually transmitted HIV infections in developing countries have been found to occur among sero-discordant couples (married or cohabiting) [25]. Disclosure of HIV results therefore provides the opportunity for women to initiate the discussion of HIV with their sexual partners. As some studies have found, this will then encourage the partners to disclose their HIV results to the women as well [11]. In this article we present findings of prevalence and factors associated with disclosure of HIV results among women in Kamwokya slum community following antenatal HIV testing.

## Methods

### Setting

Kamwokya is one of the five large slum communities in Uganda’s capital city of Kampala. The community has about 40,000 inhabitants. Most of the residents living in this slum migrated from rural areas in search of work and a better life in the urban area [26]. Male residents work mostly as petty traders, labourers and hawkers in Kampala city centre. The women are mostly engaged in petty trade, domestic work and others resort to commercial sex work. Since 1987, Kamwokya Christian Caring Community (KCCC) a local non-government organisation has provided care to those infected and affected by HIV/AIDS [26]. KCCC acts as a link between people with HIV/AIDS in the community and hospital-based health services [26]. Other HIV care organisations operating in this community include the AIDS Support Organisation (TASO) and Kamwokya Market vendors AIDS association. All these organisations provide HIV care and prevention interventions.

### Study population

We conducted a household survey between October and November 2011. The eligibility criteria included; women aged (18–45 years) who had delivered a child within 2 years prior to the study and had attended at least one antenatal care (ANC) visit during their most recent pregnancy. The antenatal care visit must have been attended at a Ministry of Health, registered health unit, and the women must have been tested for HIV during ANC.

### Recruitment of the study subjects

Kamwokya slum is divided into three administrative zones with almost equal population sizes. An equal number of the respondents were recruited from each zone and household units were used as the sampling unit. With the help of local leaders, we identified the centre of each administrative zone. At the centre of the zone, a bottle was spun on the ground and the direction in which the top of the bottle pointed was taken to be the direction of the survey. The first household selected was the first household in the direction of the survey. The second household was defined as the alternate household after the first household moving in the chosen direction. Subsequent households were selected in a similar manner and at the boundary of the zone, the interviewers turned clockwise and continued to select households until the required number of households was identified in each of the three zones.

If the woman in a selected household was not around at the time of the survey, the household was revisited the following day. If the woman was around on the following day, she was selected for the study if she met the selection criteria. If on the following day the woman was still not around, that household would be replaced by selection of another household using the procedure above.

Each research assistant had a copy of the Uganda Ministry of Health facilities inventory 2010 to cross check to ensure that the facility mentioned was a registered health facility (health centre II or of a higher level capacity).

Five hundred and sixteen (516) women were approached during the study. Of these, 501 (97.1%) consented to participate in the study; 15 women (2.9%) declined to participate because they did not have time to spare for the study. Among those who consented to participate in the study, 49 women had not delivered a child during the two year period prior to the study, 14 women had not received antenatal care during the their most recent pregnancy, 25 women did not know or had forgotten the name of the health unit where they attended ANC, and five women did not test for HIV during antenatal care. These were all excluded from the study. Therefore 408 women were eligible for inclusion in the study.

### Data collection

A standardised pre tested interviewer - administered questionnaire was used in data collection. The research assistants, both of whom had completed a Bachelors degree in Nursing, administered these questionnaires. They both underwent training on the research protocols. The data collection tools were translated into Luganda, the local language in the area. The research tools were pretested from another slum community in Kampala (Mulago Nsooba). All the information obtained was self-reported.

### Measurement of variables

To assess disclosure, we asked each woman if she had discussed her HIV test results with her partner after testing for HIV during antenatal care. The term ‘discussion’ of HIV test results was used with the aim of assessing whether a physical conversation (verbal self-disclosure) on the HIV test results occurred among the spouses. This eliminated other forms of disclosure such as non-verbal disclosure signals and assumptions.

Independent variables obtained included social demographic characteristics (age, marital status and level of education), number of antenatal care visits, having received post HIV test counselling and whether the partner tested for HIV.

Stigma was assessed using validated questions by Berger et al. [27], and those recommended by the World Health Organisation [27,28]. Four aspects of stigma were assessed, these were: the fear of transmission by casual contact, perceived/internalised stigma, enacted stigma and attitude towards disclosure of HIV results.

To assess the fear of transmission of HIV, the three questions asked were: 1) whether children with HIV should be allowed to go to school, 2) whether the individuals would buy vegetables from vendors known to have HIV, and 3) whether they would take care of their relatives if they had HIV. To assess internalised stigma, participants were asked two questions; 1) whether people with HIV should be ashamed of themselves and 2) whether people with HIV be blamed for bringing the disease to the community. To assess enacted stigma, the women were asked two questions; 1) whether they knew people with HIV who were denied health care or participation in community affairs, and 2) whether they knew people with HIV who were abused because of their HIV status. To assess the attitudes towards disclosure of HIV results, women were asked one question 1) whether they would prefer that it should remain a secret if a family member had HIV. The responses were “yes”, or “no”, or “I don’t know anyone with HIV/no opinion/not sure” for all the questions except for the questions that assessed internalised stigma for which responses were based on a 5 point likert scale. For the analysis, these responses were given a score to show the likelihood of stigma in the population based on the template scale by Bergen 2001. The three-point answers were given a score that ranged from one to two. The score was “one” if the response revealed less likelihood of stigma and “two” if the response revealed a higher likelihood of stigma. The ambiguous such as “no opinion or not sure” were scored as 1.5. Similarly questions with five-point responses were scored from one to five, with five being the responses with the most likelihood of stigma. The mean score for each type of stigma was obtained [27,28].

### Sample size

The sample size was estimated using Kish Leslie formula for descriptive studies [29]. The actual disclosure rate in the community was unknown. Therefore in order to obtain the maximum sample size, we assumed a prevalence of women’s disclosure of HIV test results in this community to be 50%. The sample size required, n, was generated as n = pqz^2^/d^2^, where p is the proportion of women who disclose their HIV results to sexual partners (p = 0.5); q = 1-p; z is 1.96 (for 5% alpha error); and d is precision which is 0.05. This gave a total of 384 women. We incorporated a non-response rate of 5% and this gave a total required study population of 405 women.

### Data analysis

Data was analysed using SPSS version 16.0 (SPSS, Chicago, IL, USA) and StatsDirect version 2.8.0 to confirm the results, and summarised using descriptive statistics. The association between the dependant variable (disclosure of HIV results to partner) and predictors was determined using bivariate analysis. The association was considered statistically significant if the p-value of the chi-square was less than 0.05. To adjust for confounding and therefore determine whether the factors were independently associated with the disclosure of HIV results, logistic regression was conducted. The plausible predictors with a statistically significant association at P ≤0.05 were entered in the logistic regression model using the rule of 10 events per variable [30]. The forward and backward regression yielded similar results and the results from the forward regression were used.

### Ethical consideration

The study was approved by the Makerere University School of Medicine Research and Ethics Committee and the Uganda National Council of Science and Technology (NCST). Verbal informed consent was obtained from all participants before recruitment into the study.

## Results

### Social demographic characteristics

The socio-demographic characteristics of the study participants are presented in Table 1. The mean age of the 408 women who participated in the study was 25.4 years (SD SD 4.94). Almost half of the women (49.5%) were below age 25 years; majority (88.4%) were Christian and had attained at least secondary level education and above (54.2%). Majority of these women (85.5%) were married/cohabiting and about two thirds were not employed outside the home (65%).

**Table 1:** **Association between socio-demographic characteristics and disclosure of HIV status with the partner**

**Characteristics**	**Total n (%)**	**Disclosed n (%) (n = 342)**	**OR (95% CI)**	**P value**
**Age**				
<25 years	202 (49.5%)	163 (80.7%)	0.63 (0.35-1.11)	0.1065
≥25 years	206 (50.5%)	179 (86.9%)		
**Religion**				
Christian	361 (88.4%)	300 (83.1%)		
Moslem	44 (10.8%)	39 (88.6%)		
Other	3 (0.8%)	3 (100%)		
**Level of education**				
Secondary and above	221 (54.2%)	201 (91%)	3.28 (1.81-6.10)	<0.0001
None/Primary	187 (45.8%)	141 (75.4%)		
**Marital status**				
Married/cohabiting	349 (85.5%)	312 (89.4%)	8.15 (4.19-15.7)	<0.0001
Never married/Widowed/separated/divorced	59 (14.5%)	30 (50.9%)		
**Occupation**				
Gainfully employed	143 (35%)	121 (84.6%)	1.00 (0.57-1.81)	0.5534
Not employed/Housewife/Student	265 (65%)	221 (83.4%)		

### Factors associated with disclosure of HIV test results

At bivariate analysis, the strongest association between the explanatory variable and the disclosure of a woman’s HIV test result was when the sexual partner had also been tested for HIV at the time when the woman was tested (OR = 30.85, CI 8.76-189.52). Attending three or more ANC visits (OR = 4.89, 95% 2.49- 9.43) and receiving post-test HIV counselling (OR = 2.88, CI 1.14 – 6.81) were also significantly associated with disclosure of HIV status to the sexual partner. Furthermore, women who had attained secondary school education and above were more likely to disclose their results to their partners compared to those who had less or no education (OR 3.28, 95% CI: 1.81-6.10). Women who were married/cohabiting were more likely to disclose their HIV results to their partners than those who were never married/widowed/separated/divorced (OR 8.15, 95% CI: 4.19-15.7). Employment status was not associated with disclosure of HIV results to the partner. These results are summarised in Table 1 & Table 2.

**Table 2:** **Predictors of disclosure of HIV test results by women**

**Characteristics**	**Total n (%)**	**n (%) disclosed to partner**	**OR (95% CI)**	**P value**
**Number of ANC visits**				
≥3 visits	347 (85%)	306 (88.2%)	5.18 (2.68-9.87)	<0.0001
≤2 visits	61 (15%)	36 (59%)		
**Received post-test counseling**				
Yes	374 (91.7%)	321 (85.8%)	3.75 (1.61-8.37)	0.001
No	34 (8.3%)	21 (61.8%)		
**Partner also tested for HIV**				
Yes	174 (42.6%)	172 (98.9%)	32.4 (9.2-198.7)	0.0001
No	234 (57.4%)	170 (72.7%)		

### Stigma levels and the association with disclosure of results to the sexual partner

The levels of stigma in this population were low and the total mean score for stigma in this population was 10.0. The mean stigma score for the women who disclosed their HIV results was 9.6 and for those who did not disclose their results, the score was 12.5 (results range from 8 to 18. Lower scores meant lower stigma levels).

With regard to whether there was an association between disclosure of HIV results and the specific aspects of stigma in this population – having either experienced or knowing someone who experienced enacted stigma, having internalised stigma or fear of transmission of HIV through casual contact- there was no difference in the mean scores between those who disclosed their HIV results to their partners and those who did not.

After logistic regression, education level, marital status, number of antenatal care visits and the preference not to disclose a family member’s HIV status remained statistically significant correlates of disclosure of HIV test results by the women interviewed. Having a partner test for HIV remained a strong predictor of disclosure of HIV test results (OR = 24.86, CI 5.30 – 116.56). However, the association between disclosure of HIV test results and having received post-test HIV counselling was not statistically significant in the multivariate analysis. These results are summarised in Table 3.

**Table 3:** **Logistic regression for predictors of disclosure of HIV test results**

**Variable**	**Adjusted odd ratio**	**95% CI**	**p – value**
Level of education (above sec vs. below sec)	2.66	1.34 - 5.30	p = 0.005
Number of ANC visits (≥3 visits vs. ≤2	3.62	1.70 - 7.72	p = 0.001
Marital status (Married/cohabiting vs. unmarried)	8.76	4.06 - 18.81	p < 0.001
Received post-test HIV counseling	2.07	0.75 - 5.71	p = 0.163
Partner also tested for HIV	24.86	5.30 - 116.56	p < 0.001
If a family member got HIV, I would want it to remain a secret	1.61	1.003 - 2.58	p = 0.049

## Discussion

This study set out to determine the prevalence and the factors associated with disclosure of HIV results determined at ANC, in a sample of women living in the slums. In this study, 83.8% of women disclosed their HIV results to their partners. This rate of HIV serostatus disclosure is comparable to disclosure rates reported in developing countries (these range from 16.7% to 86%) [1]. However, the rate of HIV disclosure reported by this study is higher than that described in other studies in Uganda. King et al. [31] reported 69% disclosure rate and Zalwango et al. [32] reported 72.6% disclosure rate [31,32]. Our disclosure rate is also higher than rates described among HIV positive women testing during ANC in Kenya where disclosure rates were found to be 52% [20]. The higher disclosure rates may have been because of differences in study populations. The three studies mentioned above were conducted among a sample of people known to be HIV positive while our study included all women who were tested at ANC regardless of their HIV serostatus. As previously reported, people who are HIV negative are more likely to disclose their HIV results than those who are HIV positive [19]. Therefore it may have been that our study sample comprised of a large percentage of women who were HIV negative. Our study limitation is that we were not able to differentiate between those who were HIV negative or positive.

Other factors previously associated with an increase in disclosure rates, and are found in this population include being married [24] and increasing time since testing (women included had been tested up to two years prior to the study) [22].

The percentage of women who disclosed their results shown by this study is encouraging. Women in the slums, as earlier mentioned, are at a greater risk of acquiring HIV than the general population and form a substantial percentage of the urban population in Uganda and other developing countries [14-17]. Therefore we hope that higher rates of disclosure of HIV results among this population results in greater access to care and improved health seeking behaviour for their sexual partners.

These study results also provide an estimate of the impact of interventions by the health care system as a whole in this high risk community.

However these results may have over/under-estimated the rate of disclosure in this population as people may have provided answers that they deemed acceptable by society [10]. Our study limitation was the failure to verify from their sexual partners on whether they actually disclosed the HIV tests.

The factors associated with disclosure of HIV results in this study population - women who were interviewed irrespective of their HIV serostatus - were mostly similar to those reported by other studies which only included women who were HIV positive.

Partner testing was strongly associated with disclosure of HIV results. This pattern followed a similar trend among those who did not disclose their HIV results in this study. Of the 66 women who did not disclose their HIV results, only 2 (3%) had their partners tested for HIV. This association was also reported by other studies [33]. The strong association observed between partner testing during ANC and disclosure of results may have been because women who test with their partners receive greater social support from them. Kizito et al. [34] reported that while most men in Uganda are aware that they can test for HIV with their partners during ANC, only 1.8% of men opt to test compared to the 62.8% of women who test for HIV [34]. Greater support has been linked to increased likelihood of disclosure of HIV results [35,36].

Disclosure of HIV results by women to their partners has been reported to be dependent on their partner’s attitude toward HIV testing. If the partner is aware and involved when the woman is testing, then the woman is more likely to disclose her HIV results [37]. Therefore, for the couples that were tested together during ANC, it may have been that they had the discussion to test for HIV prior to testing and they were both in favour of testing. Or it could mean that the women attended antenatal care with their partners and the couple was counselled together and the partner agreed to have the HIV test. This would therefore suggest that disclosure of results to partners should be viewed as a process that begins even before couple testing.

While our results indicate that couple testing is strongly associated with disclosure of HIV results, implementing couple testing has been difficult. The main hindrances have mostly been because the men were unwilling to test for HIV. The reasons being that the men who are asymptomatic do not see the need to test, and also because they view their marriages as being unstable or distrustful [38]. This is a particularly important hurdle to consider for women living in slums who reportedly have multiple sexual partners [17] and therefore unstable relationships.

Our study found that women who achieved higher education levels (secondary and above) were more likely to disclose their HIV results to their sexual partners. This finding has been found by other studies [1].

According to our study women who are married or cohabiting were more likely to disclose their HIV status. This is similar to results from other studies which also indicated that women who are married or cohabiting with their sexual partners are more likely to disclose their HIV status to the partners [4,20,21]. This may be because women who are married/cohabiting may have received greater support during the pregnancy from the partner than those who were not.

Increase in number of antenatal visits was associated with increased likelihood of disclosure of HIV results to partners. Possible reasons that may explain this are varied. Despite proximity to services, the urban poor such as women living in the slums, do not easily access required health services [39,40] and do not complete the required number of ANC visits [39]. Therefore those that had more than 3 ANC visits probably had better health seeking behaviour, or had partner support to cover the transport costs so that they are able to attend antenatal care.

Stigma and fear of discrimination have been documented in a number of studies as factors that hinder disclosure [4,12,13]. In our study, the level of stigma was low and there was no significant difference in the stigma scores observed among those who disclosed their HIV results and those who did not. Different areas in Uganda have showed varying degrees of stigma with some studies reporting reducing levels of stigma in some communities [41,42] while others reporting relatively high levels of stigma [13,43].

The low level of stigma in our study could be attributed to the increased awareness of HIV care which has been created by the government of Uganda and non governmental organisations in this community [26,44]. The lack of association of stigma with disclosure of results may show that in this particular population, disclosure of results mostly depends on other factors associated such as partner testing and not stigma. However the results may also be due to social desirability bias as people may answer based on what they think is the right thing to say rather than the truth [10].

Since disclosure is key in prevention of HIV and improving HIV care, this study has been able to shed some light concerning factors associated with disclosure of HIV results by women in the urban slums.

### Study limitations

The results from this study should be interpreted with some caution for a number of reasons. Our study was carried out in only one of the 5 large slum communities in Uganda. However, we believe that results from this community would be relatively similar to other slums in Kampala and to some extent slums in sub-Saharan Africa. This is because slum communities have a number of shared social and economic dynamics.

The study design was cross-sectional and therefore the strength of the associations may be affected by confounders that were not estimated. The information was self-reported and there was no way of verifying what was reported as the system does not have the reports; therefore this may have led to over-estimation or under-estimation of the results. There was potential for recall bias, however, only women who had delivered within the last two years were asked to participate so that they would remember over a shorter time period.

## Conclusions

Our study showed a relatively high level of disclosure of HIV test results by women to their sexual partners. Factors associated with disclosure were partner testing, number of antenatal care visits, having attained a minimum of secondary school education and marital status. To improve the rate of disclosure among women, there is need for interventions that encourage couple testing for HIV at antenatal care and encourage good healthcare seeking behaviours among pregnant women.
